# Effect of Substitution of Beef and Broiler Meat with Tuna Meat on Chemical and Sensory Quality of Meatballs

**DOI:** 10.17113/ftb.62.03.24.8278

**Published:** 2024-09

**Authors:** Nafly Comilo Tiven, Tienni Mariana Simanjorang

**Affiliations:** 1 Department of Animal Husbandry, Faculty of Agriculture, Pattimura University, Jl. Ir. M. Putuhena Kampus Unpatti-Poka, 97233 Ambon, Maluku, Indonesia; 2 Department of Agricultural Socio-Economic, Faculty of Agriculture, Pattimura University, Jl. Ir. M. Putuhena Kampus Unpatti-Poka, 97233 Ambon, Maluku, Indonesia

**Keywords:** tuna (*Thunnus* sp.) as a meat substitute, meatballs, chemical quality, cholesterol, fatty acid, sensory quality

## Abstract

**Research background:**

Tuna meat is rich in protein and polyunsaturated fatty acids (PUFA), but low in cholesterol and fat, which makes it an excellent candidate for replacing beef and broiler chicken to produce higher quality meatballs. The aim of this study is to determine how substituting beef and broiler meat with tuna meat affects the chemical and sensory characteristics of meatballs.

**Experimental approach:**

In this study, 1000 g of meatballs were prepared from 60 % of beef or broiler chicken. Each meat was replaced with tuna meat at mass fractions of 0, 20 and 40 %. The meat was finely ground and mixed with mass fractions (in %) of: tapioca flour 20, garlic 1.2, salt 2, ground pepper 0.5, egg white 0.3 and ice cubes 16. The tested variables included the chemical quality (moisture, protein, fat, ash, cholesterol and fatty acids) and sensory quality (colour, aroma, elasticity, texture and taste) of the meatballs. The data obtained were statistically analysed using a completely randomised factorial design analysis of variance.

**Results and conclusions:**

The results show that increasing the mass fraction of tuna as a substitute for beef and broiler meat significantly increased (p<0.01) the moisture, protein and PUFA mass fractions and colour, but decreased (p<0.01) the cholesterol and fat mass fraction of the meatballs. A significant interaction (p<0.01) was observed between the tuna mass fraction and the type of meat, which affected the mass fractions of moisture, protein, cholesterol and fat as well as colour of the meatballs. In particular, increasing the tuna mass fraction to 40 % significantly increased (p<0.01) the moisture mass fraction of the beef meatballs, as well as the protein mass fraction and colour of the beef and chicken meatballs. However, the moisture mass fraction of chicken meatballs and the fat and cholesterol mass fraction of beef and chicken meatballs decreased significantly (p<0.01). In conclusion, replacing 40 % of beef and chicken meatballs with tuna can improve protein content and colour, and reduce fat and cholesterol content.

**Novelty and scientific contribution:**

These results suggest that tuna can be used as a substitute for beef and chicken to produce higher quality meatballs that are rich in protein but low in cholesterol and fat. This approach can also be applied to other processed meat products such as sausages and nuggets to improve their nutritional quality.

## INTRODUCTION

Meatballs are among the most popular and well-known traditional dishes in Indonesia (*^(^**1*). Beef and chicken meatballs are processed meat products made from livestock meat (including beef, buffalo, goat, sheep, pork and poultry) mixed with starch and seasonings, with or without the addition of other permitted food ingredients, shaped into balls or other forms and then cooked (*2*). Meatballs are usually made from beef and chicken, but fish can also be used (*3*, *4*). There are several factors in favour of using fish in the production of meatballs: (*i*) economic considerations, as fish is cheaper, readily available and abundant due to Indonesia’s vast marine resources, and (*ii*) nutritional content, especially the lower cholesterol content and higher unsaturated fatty acid amounts.

The fat content of beef is approx. 7.93 % (*5*), with a cholesterol mass fractions ranging from 62 (*6*) to 85.00 mg/100 g (*7*). Chicken meat, on the other hand, contains about 5.12 % fat (*5*), which increases to 8.5 % in the breast (*6*). Cholesterol mass fraction also varies between the different parts of the chicken: breast meat ranges from 37.41 to 79.9 mg/100 g, while drumstick meat is from 48.35 to 99.5 mg/100 g (*8*). Due to this high fat and cholesterol content, meatballs made from beef and chicken can pose significant health risks, particularly due to saturated fatty acid (SFA) and cholesterol. Regular consumption of meatballs high in SFA and cholesterol can contribute to the development of atherosclerosis, which can lead to hypertension, stroke and heart attacks (*9*). It is therefore important to reduce the SFA and cholesterol content in meatballs to minimise these health risks.

Tuna (*Thunnus* sp.) is a nutritional powerhouse, rich in protein and essential unsaturated fatty acids such as omega-3, including eicosapentaenoic acid (EPA, 20:5) and docosahexaenoic acid (DHA, 22:6) (*10*), while being low in fat and cholesterol. Tuna oil contains 34.2 % ω-3 PUFA, 27.3 % DHA, 37 % EPA and 3.2 % docosapentaenoic acid (DPA) (*9*). The crude protein content of yellowfin tuna is 23.52 % and that of bigeye tuna is 23.72 %, with a crude fat content of 1.93 and 2.06 %, respectively. The DHA content of these species is 16.91 and 20.22 %, while the EPA content is 2.39 and 3.27 % (*11*). The cholesterol mass fraction of tuna varies greatly and is between 13 and 60 mg/100 g depending on the type of tuna and the method of preparation (*12*).

Substituting beef and chicken with tuna (*Thunnus* sp.) in the production of meatballs has not been previously reported. Existing studies have investigated the improvement of beef meatballs with milkfish (*Channos channos* Forsk) (*13*) and cork fish (*14*). Therefore, this study investigates tuna meat as a substitute for beef and chicken to produce high-quality meatballs that are rich in protein and essential unsaturated fatty acids but low in cholesterol and have better sensory properties. The aim of this study is to determine the chemical quality, cholesterol content, fatty acid composition and sensory properties of beef and chicken meatballs substituted with tuna (*Thunnus* sp.). The results will contribute to the development of high-quality meatballs as supplementary food for school children and thus possibly help to prevent stunting.

## MATERIALS AND METHODS

### Materials

The materials used in this study included livestock meat: beef (*biceps femoris* muscle), broiler breast (*pectoralis* muscle) and tuna fillet. Other ingredients were commercial tapioca flour (Rose Brand) produced by Budi Acid Jaya, Lampung, Indonesia, seasonings (salt, garlic and ground pepper), ice cubes and egg white as a binder. Beef and chicken were purchased from local meat markets, tuna fillets from fish markets and flour and seasonings from grocery shops, all located in the traditional market of Mardika in Ambon City, Maluku Province, Indonesia. The equipment used in this study included an electric meat grinder (Willman MG30, Jakarta, Indonesia), a chopper (Mitochiba CH-200, Jakarta, Indonesia), a cooker (Hock, Jakarta, Indonesia) and a 28-cm boiling pot (Orchid, Jakarta, Indonesia). All appliances were purchased from a department store in the Mardika shopping area, Ambon City, Maluku Province. Additionally, an analytical balance (EX224/AD Ohaus, Jakarta, Indonesia) was used to weigh samples and a gas chromatograph (GC) (Agilent Technologies 7890B, Santa Clara, CA, USA) was used to analyse the fatty acid content.

### Meatball processing

The meatballs were prepared with the following ingredients (in *w*/%): meat 60, salt 2, tapioca flour 20, garlic 1.2, ground pepper 0.5, egg white 0.3 and ice cubes 16. Beef, chicken and tuna were each ground to a fine texture. The livestock meat was replaced with tuna at mass fractions of 0, 20 and 40 %. The ground meat was mixed with the prepared seasonings, tapioca flour and ice cubes and then finely ground to obtain a homogeneous mixture. This mixture was shaped into balls 2 cm in diameter and cooked in boiling water in separate batches according to the treatment groups until fully cooked, *i.e* until the meatballs floated. The cooked meatballs were then removed, drained and prepared for subsequent analysis.

### Chemical quality analysis

Before the meatballs were prepared, the chemical quality of the beef, chicken and tuna fillets was analysed. After the meatballs were prepared, their chemical quality was analysed again and mass fractions of moisture, protein, fat and ash were determined (*15*).

### Cholesterol analysis

The cholesterol mass fraction of beef, chicken and tuna was measured both before and after the production of meatballs (*15*). A mass of 1 g of sample was weighed and transferred to an Erlenmeyer flask to which 2 mL of 50 % KOH were added. The mixture was vortexed to ensure homogenisation and then 95 % ethanol was added. The sample was saponified for 15 min at 80–100 °C. After saponification, the mixture was cooled with water and 10 mL of toluene were added. The solution was stirred for 10 s and then transferred to a separating funnel. Then, 10 mL of 1 M KOH and 1 mL of 95 % ethanol were added. The two layers were separated and the lower layer was carefully discarded. This washing step was repeated twice with the addition of 10 mL of distilled water and the lower layer was removed each time. The final mixture was transferred to a vacuum tube and a 1 µL aliquot was injected into a gas chromatograph. After analysis, the chromatogram was recorded and the resulting curve was compared to a standard cholesterol reference for quantification.

### Fatty acid composition analysis

#### Hydrolysis

A mass of 5 g of sample was weighed and placed in a large test tube. Then, 10 mL of saturated HCl were added and the mixture was heated in a water bath at 80 °C for 3 h. After cooling, the mixture was extracted with 25 mL of a mixture of diethyl ether and petroleum ether (1:1). The solution was vortexed and allowed to stand until the layers separated, after which the upper layer, containing the oil, was collected. The oil was then evaporated in a water bath using N_2_ gas.

#### Methylation

A volume of 0.5 mL aliquot of the extracted oil was placed in a small, closed test tube. Then, 1.5 mL of a methanolic sodium chloride solution was added. The tube was covered and heated at 60 °C for 5–10 min with shaking. After cooling, 2 mL of boron trifluoride methanoate were added and the mixture was heated again at 60 °C for 5–10 min. After cooling, the solution was extracted with 1 mL of heptane and 1 mL of saturated NaCl. The upper layer was then collected and placed in a vial for injection into the gas chromatograph (GC).

#### GC analysis

The conditions for the analysis using Agilent Technologies 7890B gas chromatograph were as follows: injection volume 1 µL, temperature 26 °C, pressure 47.914 Pa, total flow 22.25 mL/min, column flow 1.75 mL/min, purge flow 3 mL/min, split ratio 10:1 and split flow 17.5 mL/min. Detector temperature was 260 °C, carrier gas He, makeup gas N_2_, makeup flow 30 mL/min, H_2_ flow 40 mL/min and airflow 400 mL/min. Column (HP-88) specifications were as follows: length 100 m, inner diameter 0.25 mm, film thickness 0.2 µm. The resulting chromatograms were compared with standard chromatograms to identify and quantify fatty acids.

### *Evaluation* of *sensory properties*

The sensory properties were evaluated in a laboratory setting with 25 untrained panellists consisting of 15 females and 10 males aged 21–22, all from the Department of Animal Husbandry, College of Animal Product Technology, Faculty of Agriculture, Pattimura University, Ambon, Indonesia. The evaluation took place at 15:00 h in a room with a temperature of 24 °C. The sensory attributes (colour, aroma, elasticity, texture and taste) of the raw materials (beef, chicken and tuna) used for making meatballs and the beef and chicken meatballs substituted with tuna were evaluated and the scores are shown in Table 1.

**Table 1:** Scores and sensory properties of raw materials and meatballs with partial replacement of meat with tuna

Score	Colour	Aroma	Elasticity	Texture	Taste
1	Very pale/Very white	Very fishy/Very stinky	Not very chewy	Very rough	Not very tasty
2	Pale/White	Fishy/Stinky	Not chewy	Rough	Not tasty
3	Slightly bright red/Slightly grey	Slightly fresh/Slightly smells like meat	A bit chewy	Rather smooth	Rather tasty
4	Bright red/Grey	Fresh/Smells like meat	Chewy	Smooth	Tasty
5	Purplish red/Very grey	Very fresh/Smells very much like meat	Very chewy	Very smooth	Very tasty

Samples of the raw materials and the meatballs with varying mass fractions of tuna were placed on labelled test plates and served in a random order. Each panellist received three test plates with the raw materials and three test plates with meatballs containing tuna meat substitutes, depending on the treatment. Panellists were given a glass of mineral water to rinse off between samples and neutralise the aftertaste. They were instructed to observe, smell, hold/touch and taste each sample and then complete a questionnaire on the attributes listed in Table 1.

### Statistical analysis

Data on the raw materials used for the production of meatballs were analysed using analysis of variance (ANOVA) with a completely randomised design including three types of meat (beef, broiler and tuna), with five replicates each. The meatball data were analysed using ANOVA with a completely randomised factorial design, with factor A (two types of livestock meat: beef and broiler) and factor B (three mass fractions of tuna: 0, 20 and 40 %), with each treatment replicated five times. The differences between treatments were further analysed using Duncan’s multiple range test (*16*). Statistical analysis was conducted using IBM Statistic SPSS v. 26 for Windows (*17*).

## RESULTS AND DISCUSSION

### Chemical composition of raw materials

The chemical composition of the raw materials used for the meatballs (beef, chicken and tuna) is shown in Table 2. Statistical analysis revealed significant differences (p<0.01) among the raw materials (beef, chicken and tuna) in the mass fraction of water, fat and ash. Protein mass fraction was not significantly different.

**Table 2:** Chemical quality of beef, broiler and tuna used for the preparation of meatballs

Parameter	Beef	Chicken	Tuna
*w*(moisture)/%	(77.1±1.1)^a^	(76.3±0.6)^a^	(73.3±0.3)^b^
*w*(protein)/%	(23.9±0.1)	(23.7±0.3)	(23.7±0.3)
*w*(fat)/%	(0.9±0.0)^b^	(1.2±0.1)^a^	(0.28±0.0)^c^
*w*(ash)/%	(0.4±0.0)^ab^	(0.5±0.0)^a^	(0.4±0.0)^b^
*w*(cholesterol)/(mg/100 g)	(16.9±1.6)^a^	(15.6±1.8)^a^	(11.5±0.9)^b^

Mean values in the same row with different letters in superscript differ significantly (p<0.01). Results are expressed as mean value±standard deviation, *N*=5

The moisture mass fraction of the raw materials varied between 73.2 and 77.1 %. Beef had the highest moisture content at 77.1 %, which was not significantly different from that of chicken at 76.3 %, but was significantly higher (p<0.01) than that of tuna at 73.2 %, which had the lowest moisture mass fraction. Fat mass fraction ranged from 0.28 to 1.18 %, with chicken having the highest fat mass fraction at 1.18 %, significantly higher (p<0.01) than beef (0.92 %) and tuna (0.28 %), the latter having the lowest fat mass fraction. In addition, the fat content of beef was significantly higher (p<0.01) than that of tuna. The ash mass fractions ranged from 0.42 to 0.47 %, with chicken again having the highest value at 0.47 %, which was significantly higher (p<0.01) than that of beef (0.44 %) and tuna (0.42 %), the latter being the lowest. Additionally, the ash mass fraction of beef was significantly higher (p<0.01) than that of tuna.

The moisture content of beef and chicken was higher than that of tuna due to the high fat content of beef and chicken, which leads to an increased intramuscular fat content (marbling). This marbling loosens the microstructure of the muscle fibres and provides ample space for the meat proteins to bind water. According to Amertaningtyas (*18*), the average moisture mass fraction of fresh beef was 76.53 %, while Arizona *et al.* (*19*) reported an average of 76.04 % in *longissimus dorsi* muscle. The moisture and protein content of broiler chicken in this study was higher than that of Nuraini *et al*. (*20*), who found mass fractions of moisture 74.33 % and protein 17.75 % in broiler chicken.

Chicken meat had a higher fat and ash content than beef and tuna. However, the fat and ash content of broiler chicken in this study was still lower than that of Rukmini *et al.* (*21*), who found fat and ash mass fractions of 2.54 to 2.72 % and 1.61 to 1.72 %, respectively, in broiler chicken with different cage densities. According to Milićević *et al.* (*8*), chicken breast meat contains 70.74–74.29 % water, 21.18–22.29 % protein, 2.61–5.53 % fat and 0.99–1.29 % ash. The low fat content of the chicken meat used in this study could therefore be related to the high moisture content of the meat, as fat content is negatively correlated with moisture content. Additionally, the chicken meat used in making meatballs was breast meat from which the fat had been removed.

The moisture mass fraction of frozen tuna loin was reported to be 72.57 % (*22*). The red meat of the tuna contained 24.67 % protein, 0.92 % fat and 1.82 % ash (*23*). Peng *et al.* (*11*) reported that the fin meat of yellowfin tuna contained 73.57 % moisture, 23.52 % protein, 1.93 % fat and 1.54 % ash. In addition, Suseno (*23*) documented the chemical composition of tuna red meat by-products as 58.56 % moisture, 24.67 % protein, 0.92 % fat and 1.82 % ash.

### Cholesterol content of raw materials

Table 2 shows the cholesterol content of the raw materials (beef, broiler chicken and tuna) used for the production of meatballs. Statistical analysis revealed significant differences in cholesterol content among these raw materials (p<0.01). The cholesterol mass fractions ranged from 11.5 to 16.9 mg/100 g. Beef had the highest cholesterol content at 16.9 mg/100 g, which was not significantly different from chicken meat at 15.6 mg/100 g, but was significantly higher (p<0.01) than tuna meat, which had the lowest cholesterol mass fraction at 11.5 mg/100 g.

The cholesterol content of beef in this study is comparable to the findings of Yulianto and Bulkaini (*24*), who reported that the cholesterol mass fraction of Bali beef was between 16.38 and 17.25 mg/100 g. The cholesterol mass fraction of broiler chicken in this study is lower than the results of Milićević *et al.* (*8*), who found that the cholesterol mass fraction of chicken breast and drumstick ranged from 37.41 to 79.9 mg/100 g and 48.35 to 99.5 mg/100 g, respectively. The low cholesterol content in chicken could be due to the release of fat tissue during the production of meatballs. Additionally, the low cholesterol could be due to the young age of the chicken, as the low-density lipoprotein (LDL) receptors can decrease with age, causing LDL levels in the blood to rise and clog blood vessels (*24*). The use of tuna as a substitute for beef and chicken in meatballs is highly appropriate due to its lower fat and cholesterol content, while its protein content is comparable to that of beef and chicken.

### Fatty acid composition of raw materials

The fatty acid composition of the raw meat materials (beef, chicken and tuna) used for the production of meatballs is shown in Table 3. Statistical analysis revealed significant differences (p<0.01) in mass fractions of monounsaturated fatty acids (MUFA), polyunsaturated fatty acids (PUFA), ω-3 and ω-9, but no significant differences in saturated fatty acids (SFA) and ω-6 among the raw meat materials.

**Table 3:** Fatty acid composition of raw meat used for the preparation of meatballs

		w(fatty acid)/%	
Fatty acid	Beef	Chicken	Tuna
C6:0	(0.0±0.0)	(0.2±0.0)	(0.00±0.0)
C12:0	(0.1±0.0)	(0.7±0.2)	(1.24±0.0)
C14:0	(1.7±0.0)	(0.9±0.5)	(1.8±1.0)
C15:0	(0.4±0.1)	(0.2±0.0)	(0.0±0.0)
C15:1	(0.5±0.0)	(0.0±0.0)	(0.0±0.0)
C16:0	(0.9±0.0)	(28.5±0.0)	(0.0±0.0)
C16:1	(26.0±0.2)	(16.9±14.5)	(27.1±2.3)
C17:0	(1.4±0.1)^b^	(4.4±0.6)^a^	(1.8±0.2)^b^
C17:1	(1.4±0.1)^a^	(0.0±0.0)^b^	(0.0±0.0)^b^
C18:0	(1.0±0.0)	(0.3±0.0)	(0.0±0.0)
C18:1 ω-9	(25.7±2.0)^a^	(9.2±2.5)	(18.2±3.9)^b^
C18:2 ω-6	(30.6±0.4)^b^	(40.5±2.6)^a^	(29.0±1.4)^b^
C18:3 ω-6	(6.9 ±0.5)^ab^	(13.5±6.9)^a^	(4.8 ±0.8)^ab^
C20:1 ω-9	(1.3±0.0)	(0.0±0.0)	(0.0±0.0)
C21:0	(0.8±0.6)^a^	(0.5±0.3)^ab^	(0.0±0.0)^c^
C20:2 ω-6	(0.0±0.0)	(0.3±0.1)	(0.0±0.0)
C22:0	(0.5±0.0)	(1.8±1.2)	(0.0±0.0)
C22:1 ω-9	(0.7±0.0)	(0.4±0.0)	(0.0±0.0)
C23:0	(1.6±0.0)^b^	(1.7±0.8)^b^	(3.7±1.0)^a^
C20:5 ω-3	(0.0±0.0)	(0.1±0.0)	(0.00±0.00)
C24:0	(0.4±0.0)^b^	(0.3±0.1)^b^	(1.8±0.6)^a^
C24:1 ω-9	(0.0±0.0)^b^	(0.5±0.2)^a^	(0.0±0.0)^b^
C22:6 ω-3	(0.9±0.1)^b^	(0.3±0.2)^b^	(11.4±6.3)^a^
SFA	(7.4±1.0)	(18.7±16.2)	(9.5±1.6)
MUFA	(54.2±1.2)^a^	(26.7±12.1)^b^	(45.3±6.1)^a^
PUFA	(38.4±0.3)^b^	(54.7±4.2)^a^	(45.2±4.8)^b^
ω-3	(1.8±1.0)^c^	(4.8±1.0)^b^	(7.8±1.0)^a^
ω-6	(6.9±0.5)	(6.1±6.7)	(4.8±0.8)
ω-9	(26.6±1.2)^a^	(9.8±2.4)^c^	(18.2±3.9)^b^

Mean values in the same row with different letters in superscript differ significantly (p<0.01). Results are expressed as mean value±standard deviation, *N*=5

The MUFA content of the raw materials ranged from 26.7 to 54.2 %, with beef having the highest MUFA content (54.2 %), which was not significantly different from tuna (45.3 %), but significantly higher (p<0.01) than that of chicken (26.7 %). Tuna also had a significantly higher MUFA content (p<0.01) than chicken. The PUFA content ranged from 38.4 to 54.7 %, with chicken having the highest PUFA content (54.7 %), which was significantly higher (p<0.01) than that of beef (38.4 %) and tuna (45.2 %). Beef and tuna did not differ significantly in PUFA content.

The ω-3 content ranged from 1.8 to 7.8 %, with tuna having the highest ω-3 content (7.8 %), significantly higher (p<0.01) than chicken (4.8 %) and beef (1.8 %). Chicken also had a significantly higher ω-3 content (p<0.01) than beef. The ω-9 content ranged from 9.8 to 26.6 %, with beef having the highest ω-9 content (26.6 %), significantly higher (p<0.01) than tuna (18.2 %) and chicken (9.8 %). Tuna also had a significantly higher ω-9 content (p<0.01) than chicken.

A total of 23 types of fatty acids were detected in the raw meat materials. Chicken had less MUFA (p<0.01) but more PUFA (p<0.01) than beef and tuna. The high MUFA mass fraction in beef is due to the high mass fraction of C18:1 ω-9 (methyl *cis*-9-oleate), which Lukic *et al.* (*25*) identified as the predominant MUFA in beef. The high PUFA mass fraction of chicken is due to significant amounts (p<0.01) of C18:2 (methyl linolelaidate) and C18:3 ω-6 (γ-linolenic acid methyl ester). The PUFA mass fraction in chicken observed in this study is higher than that reported by Milićević *et al.* (*8*), who found a PUFA mass fraction of 24.25 to 25.46 % in chicken breast with C18:2 and C18:3 ω-6 accounting for only 0.19 to 0.22 %.

The high ω-3 mass fraction (p<0.01) in tuna compared to chicken and beef is due to the significant presence of C22:6 ω-3 (methyl *cis*-4,7,10,13,16,19-docosahexaenoic acid) (DHA). The C22:6 ω-3 mass fraction in this study exceeds the value reported by Suseno (*22*), who found 8.82 % C22:6 ω-3 in tuna by-products. The high ω-9 mass fraction (p<0.01) in beef compared to tuna and chicken is due to the high content of C18:1 ω-9 (methyl *cis*-9-oleate), which is consistent with Lukic *et al.* (*25*).

Although the difference was not significant, chicken had a higher SFA content than beef and tuna due to higher mass fractions of C16:0 (methyl palmitate) and C17:0 (methyl heptadecanoate). For example, the chicken used in this study contained 28.51 % C16:0, while Milićević *et al.* (*8*) reported C16:0 mass fractions in chicken breast ranging from 22.26 to 26.81 %.

### Sensory properties of raw materials

Table 4 shows the sensory properties of the raw materials (beef, chicken and tuna) used for the production of meatballs. Statistical analysis revealed significant differences (p<0.01) in colour and texture of the meat, while there were no significant differences in aroma and tenderness. Beef, with a score of 3.1 (slightly bright red), had a significantly higher (p<0.01) colour score than broiler (2.3, pale) and tuna (2.1, pale). There was no significant difference in colour between broiler and tuna. The texture of tuna, with a score of 4.1 (smooth), differs very significantly (p<0.01) from the texture of beef, with a score of 3.3 (slightly smooth) and the texture of broiler with a score of 3.1 (slightly smooth). The texture of beef is not significantly different from the texture of broiler. Tuna, with a texture score of 4.1 (smooth), was significantly different (p<0.01) from beef (3.3, slightly smooth) and broiler (3.1, slightly smooth). The texture of beef did not significantly differ from that of broiler.

**Table 4:** Sensory properties of raw meat samples used for the preparation of meatballs

Parameter	Beef	Chicken	Tuna
Colour	(3.1±0.9)^a^	(2.3±0.7)^b^	(2.1±0.8)^b^
Aroma	(3.7±1.2)	(3.1±0.7)	(3.7±0.7)
Elasticity	(3.8±0.6)	(3.3±0.6)	(3.5±0.7)
Texture	(3.3±1.0)^b^	(3.1±1.0)^b^	(4.1±0.6)^a^

Mean values in the same row with different letters in superscript differ significantly (p<0.01). Results are expressed as mean value±standard deviation, *N*=5

The colour of meat plays an important role in the visual stimulation of consumers and influences their willingness to purchase or reject a meat product. Various intrinsic factors (such as final pH, age of the animal, muscle position, breed, slaughter mass and sex) and extrinsic factors (such as production systems and feeding, pre-slaughter stress, slaughter season and chilling rates) influence meat colour (*26*). The red colour of the meat is particularly important for consumer evaluation, as fresh red meat is generally preferred to purple or brown meat (*27*).

#### Properties of beef

The beef used in this study had a bright red colour, with an average score of 3.1 (Table 4). This observation is consistent with Merthayasa *et al.* (*28*), who reported that Bali beef typically has a bright red colour due to exposure to oxygen, which oxidises myoglobin to oxymyoglobin. The beef colour score in this study exceeded that of Liur *et al.* (*29*), who found the highest value for beef colour in various shops in Ambon City, Indonesia, to be 2.85.

Texture refers to the sense of touch and includes properties such as hardness, roughness or smoothness. The texture of beef is determined by factors such as water and fat content. In this study, the beef had a relatively smooth texture, with an average score of 3.3. The texture of beef is influenced by its fat content and muscle fibres. While fat marbling can improve smoothness, the presence of slightly coarse muscle fibres can affect the overall fineness of the meat texture. These results are consistent with those of Liur *et al.* (*29*), who reported the highest texture score for beef in different shops in Ambon City to be 3.00.

#### Properties of broiler meat

In this study, the broiler meat had a pale colour, with an average score of 2.3 (Table 4). This finding is in agreement with that of Hajrawati *et al.* (*30*), who found that broiler meat in traditional markets in Bogor generally has a pale colour, scoring around 3.00. The location of the muscles can affect the colour of chicken meat, and in this study, we used breast meat, which is known to be paler than other parts of the chicken. Wideman *et al.* (*31*) reported that chicken legs and thighs are darker than breast meat due to their higher content of myoglobin and haem pigments.

The texture of the chicken meat used in this study was relatively smooth with an average score of 3.1. These results are consistent with those of Hajrawati *et al.* (*30*), who found that the texture of chicken meat in traditional markets in Bogor ranges from 3.00 (rather rough) to 4.00 (soft, tender).

#### Properties of tuna meat

In this study, the tuna meat had a pale colour, with an average score of 2.1. Loppies *et al.* (*32*) reported that tuna muscle typically displays a bright red colour, which is vital for its market value because consumers often associate red tuna meat with freshness. However, the colour of tuna flesh can vary depending on the species, with some species having naturally paler flesh due to exposure to oxygen. *Euthynnus affinis*, commonly known as little tunny or kawakawa, for example, has a lighter flesh with an L* value of 38.72 (*33*). The L* value stands for the lightness of the flesh and ranges from 0 (black) to 100 (white). The pale colour observed in tuna meat can be attributed to oxidation processes in which cations convert Fe^2+^ in myoglobin to Fe^3+^ and thus change the colour of the meat (*32*).

The texture of the tuna meat was found to be smooth with an average score of 4.1 (Table 4). This smooth texture is probably due to the finer fibre structure of tuna meat than that of beef and chicken. In addition, the relatively low mass fraction of SFA and the high mass fraction of MUFA and PUFA in tuna meat contribute to its smooth texture.

### Chemical quality of meatballs substituted by tuna meat

Table 5 shows the chemical quality of beef and chicken meatballs with different mass fractions of tuna substitution. Statistical analysis showed that both the type of meat and the mass fraction of tuna substitution significantly affected (p<0.01) the moisture, protein and fat content of the meatballs. Although the type of meat did not significantly affect the ash content, the mass fraction of tuna had a very significant effect (p<0.01) on the ash content. A significant interaction between the type of meat and the mass fraction of tuna meat was observed for the moisture, protein and fat content, but not for the ash content. Beef meatballs had higher moisture and protein mass fractions (p<0.01), but a lower fat content (p<0.01) than chicken meatballs. Increasing the amount of tuna increased protein mass fraction (p<0.01), while the effects on moisture and fat content were inconsistent.

**Table 5:** Chemical quality of meatballs with partial replacement of meat with tuna

		*w*(tuna)/%			
Parameter	Meat	0	20	40	Average
*w*(moisture)/%	Beef	(67.5±0.1)^E^	(68.1±0.0)^D^	(68.4±0.2)^D^	(68.2±0.5)^a^
	Chicken	(68.1±0.5)^D^	(68.1±0.2)^D^	(64.3±0.3)^F^	(67.3±1.7)^b^
	Average	(67.8±0.4)^b^	(68.1±0.0)^a^	(66.4±2.9)^c^	
*w*(protein)/%	Beef	(15.0±0.1)^E^	(14.4±0.1)^F^	(15.6±0.4)^D^	(14.8±0.5)^a^
	Chicken	(11.9±0.2)^G^	(12.1±0.4)^G^	(14.2±0.1)^F^	(12.8±0.9)^b^
	Average	(13.4±2.2)^b^	(13.2±1.6)^b^	(14.9±1.0)^a^	
*w*(fat)/%	Beef	(0.6±0.0)^F^	(0.6±0.0)^G^	(0.6±0.1)^G^	(0.6±0.0)^b^
	Chicken	(0.9±0.0)^E^	(0.9±0.0)^E^	(1.0±0.0)^D^	(1.0±0.1)^a^
	Average	(0.8±0.2)^a^	(0.7±0.2)^b^	(0.8±0.3)^a^	
*w*(ash)/%	Beef	(0.6±0.0)	(0.5±0.0)	(0.6±0.0)	(0.6±0.0)
	Chicken	(0.6±0.0)	(0.5±0.0)	(0.6±0.0)	(0.6±0.0)
	Average	(0.6±0.0)^a^	(0.5±0.0)^b^	(0.6±0.0)^a^	
*w*(cholesterol)/(mg/100 g)	Beef	(21.1±1.9)^F^	(12.2±0.3)^G^	(13.8±1.4)^G^	(14.3±6.8)^b^
	Chicken	(34.6±2.4)^D^	(24.7±1.1)^E^	(13.2±0.1)^G^	(21.6±9.4)^a^
	Average	(0.6±0.0)^a^	(0.5±0.0)^b^	(0.6±0.0)^a^	

Mean values in the same column/row with different lowercase letters in superscript differ significantly (p<0.01) within individual treatments. Mean values in the same column/row with different uppercase letters in superscript indicate significant differences due to interaction effects between types of meat and mass fractions of tuna as a meat substitute (p<0.01). Results are expressed as mean value±standard deviation, *N*=5

Increasing the tuna mass fractions resulted in a higher protein content, but caused variations in the moisture, fat and ash content of the meatballs. These results are consistent with the NSC guidelines (*2*), which specify mass fractions: maximum for moisture 70 %, minimum for protein 8 %, maximum for fat 10 % and maximum for ash 3 %.

There was a highly significant interaction (p<0.01) between the type of meat and the mass fraction of tuna in relation to the moisture, protein and fat mass fractions of the meatballs. The addition up to 40 % of tuna significantly (p<0.01) increased the protein content of beef meatballs and also the fat content of chicken meatballs (p<0.01). The increase in protein content in beef meatballs is due to the additional protein from the tuna. However, this effect was not observed in chicken meatballs, which have a higher fat content than beef (Table 2). This interaction also has an effect on the moisture content of the meatballs: increasing the tuna mass fraction up to 40 % significantly increased (p<0.01) the moisture content of the beef meatballs, but decreased (p<0.01) the moisture content of the chicken meatballs. This can be explained by the water-holding capacity (WHC) of meat proteins, where an increase in protein content generally increases the WHC and thus increases the moisture content of the meatballs. This result is consistent with Fillaili *et al.* (*34*), who found that a higher protein content in tofu residues improved the WHC and increased the moisture content of tilapia meatballs.

### The cholesterol in meatballs replaced by tuna meat

Table 5 shows the cholesterol content of meatballs made from beef and chicken with replacement of meat with different mass fractions of tuna. The statistical analysis showed that both the type of meat and the mass fraction of tuna significantly (p<0.01) affected the cholesterol content of the meatballs. Beef meatballs had a lower cholesterol content (p<0.01) than chicken meatballs. Increasing the mass fraction of tuna led to a significant decrease (p<0.01) in cholesterol content. The cholesterol mass fraction of meatballs without tuna (0 %) was 27.9 mg/100 g and was therefore significantly higher (p<0.01) than of meatballs with 20 % tuna (18.4 mg/100 g) and 40 % tuna (13.5 mg/100 g). The cholesterol content in meatballs with 20 % tuna was also higher (p<0.01) than of those with 40 % tuna.

Compared to meatballs without tuna (0 %), the chicken meatballs with tuna substitute showed a significant reduction in cholesterol mass fractions. This reduction is due to the lower cholesterol mass fraction of tuna meat (Table 2) and its high mass fractions of C18:3 ω-6 and C22:6 ω-3 (DHA) (Table 3), which contribute to the reduction of cholesterol and triglyceride mass fractions in the meatballs.

### The fatty acid composition of meatballs with tuna substitute

Table 6 shows the fatty acid composition of beef and chicken meatballs with different mass fractions of tuna as substitute. Statistical analysis showed that both the type of meat and the mass fraction of tuna as substitute had a significant effect (p<0.01) on the SFA, MUFA, PUFA, ω-3, ω-6 and ω-9 content of the meatballs. Beef meatballs with tuna substitutes had higher mass fractions (p<0.01) of MUFA, ω-3, ω-6 and ω-9, while chicken meatballs partially replaced with tuna had higher mass fractions (p<0.01) of SFA and PUFA. A significant interaction (p<0.01) was found between the type of meat and the amount of tuna, which had an effect on the fatty acids C8:0, C13:0, C15:1, C18:0, C18:3 ω-6, C20:1 ω-9, C21:0, C22:0 and C24:0.

**Table 6:** Fatty acid composition of meatballs with partial replacement of meat with tuna

		*w*(tuna)/%			
Fatty acid	Meat	0	20	40	Average
			*w*(fatty acid)/%		
C4:0	Beef	(0.1±0.1)	(0.1±0.1)	(0.0±0.0)	(0.1±0.1)
	Chicken	(1.0±1.6)	(1.2±2.1)	(0.6±0.9)	(0.9±0.3)
	Average	(0.6±1.2)	(0.7±1.4)	(0.3±0.7)	
C6:0	Beef	(0.1±0.2)	(0.2±0.2)	(0.1±0.1)	(0.1±0.1)
	Chicken	(0.2±0.1)	(0.0±0.1)	(0.0±0.0)	(0.1±0.1)
	Average	(0.2±0.2)	(0.1±0.2)	(0.0±0.1)	
C8:0	Beef	(0.7±0.3)^D^	(0.0±0.0)^E^	(0.0±0.0)^E^	(0.2±0.4)^a^
	Chicken	(0.1±0.1)^E^	(0.0±0.1)^E^	(0.0±0.0)^E^	(0.0±0.0)^b^
	Average	(0.4±0.4)^a^	(0.0±0.0)^b^	(0.0±0.0)^b^	
C10:0	Beef	(0.2±0.4)	(0.1±0.2)	(0.1±0.0)	(0.1±0.1)
	Chicken	(0.0±0.0)	(0.0±0.0)	(0.0±0.0)	(0.0±0.0)
	Average	(0.1±0.3)	(0.1±0.1)	(0.1±0.1)	
C12:0	Beef	(2.4±3.7)	(1.4±0.8)	(0.80±0.3)	(1.4±0.9)
	Chicken	(1.0±0.1)	(0.8±0.1)	(0.9±0.03)	(0.9±0.1)
	Average	(1.7±2.1)	(0.9±0.5)	(0.9±0.2)	
C13:0	Beef	(0.7±0.4)^D^	(0.2±0.2)^E^	(0.0±0.0)^E^	(0.3±0.3)^a^
	Chicken	(0.0±0.0)^E^	(0.00±0.0)^E^	(0.0±0.0)^E^	(0.0±0.0)^b^
	Average	(0.3±0.4)^a^	(0.1±0.2)^b^	(0.0±0.0)^b^	
C14:0	Beef	(2.6±2.7)	(1.3±1.2)	(1.7±1.0)	(1.9±0.6)
	Chicken	(1.5±0.2)	(1.8±0.1)	(1.3±0.1)	(1.3±0.2)
	Average	(2.0±1.8)	(1.3±0.7)	(1.5±0.7)	
C15:0	Beef	(0.0±0.0)	(0.0±0.0)	(0.4±0.5)	(0.1±0.2)
	Chicken	(0.1±0.1)	(0.2±0.0)	(0.2±0.0)	(0.2±0.1)
	Average	(0.0±0.1)^b^	(0.1 ±0.1)^ab^	(0.3±0.3)^a^	
C15:1	Beef	(0.0±0.0)^E^	(0.0±0.0)^E^	(1.4±0.3)^D^	(0.4±0.8)^a^
	Chicken	(0.0±0.0)^E^	(0.2±0.3)^E^	(0.05±0.08)^E^	(0.1±0.1)^b^
	Average	(0.0±0.0)^b^	(0.1±0.2)^b^	(0.7±0.8)^a^	
C16:0	Beef	(0.0±0.0)	(0.0±0.0)	(0.0±0.0)	(0.00±0.00)^b^
	Chicken	(30.0±3.3)	(18.9±16.4)	(9.4±16.2)	(19.4±10.3)^a^
	Average	(15.0±16.6)	(9.5±14.7)	(4.7±11.5)	
C16:1	Beef	(23.9±1.7)	(24.4±1.2)	(24.1±2.8)	(24.1±0.2)^a^
	Chicken	(0.3±0.0)	(9.1±15.4)	(17.7±15.2)	(9.0±8.7)^b^
	Average	(12.1±13.0)	(16.8±12.9)	(20.9±10.4)	
C17:0	Beef	(2.8±0.2)	(2.8±0.7)	(2.8±1.1)	(2.8±0.0)^b^
	Chicken	(6.1±0.6)	(5.4±0.3)	(5.9±0.1)	(5.8±0.4)^a^
	Average	(4.5±1.8)	(4.1±1.5)	(4.4±1.8)	
C17:1	Beef	(0.0±0.0)^E^	(0.0±0.0)^E^	(0.90.4)^D^	(0.3±0.5)
	Chicken	(0.1±0.1)^E^	(0.1±0.1)^E^	(0.2±0.2)^E^	(0.1 ±0.1)
	Average	(0.1±0.1)^b^	(0.0±0.1)^b^	(0.6±0.5)^a^	
C18:0	Beef	(0.0±0.0)^E^	(0.0±0.0)^E^	(1.0±0.3)^D^	(0.3±0.6)^a^
	Chicken	(0.0±0.1)^E^	(0.1±0.0)^E^	(0.1±0.1)^E^	(0.1±0.1)^b^
	Average	(0.0±0.1)^b^	(0.1±0.1)^b^	(0.5±0.5)^a^	
C18:1 ω-9	Beef	(13.7±3.1)	(10.3±3.9)	(14.3±7.5)	(12.7±2.2)^a^
	Chicken	(8.4±0.6)	(8.0±1.2)	(7.3±0.5)	(7.9±0.6)^b^
	Average	(11.0±3.5)	(9.2±2.8)	(10.8±6.1)	
C18:2 ω-6	Beef	(25.4±0.2)^F^	(25.2±4.5)^F^	(30.1±1.7)^E^	(26.9±2.8)^b^
	Chicken	(43.8±3.7)^D^	(43.6±1.2)^D^	(40.5±0.5)^D^	(42.6±1.8)^a^
	Average	(34.6±10.3)	(34.4±10.5)	(35.3±5.8)	
C18:3 ω-6	Beef	(13.7±0.2)^DE^	(15.6±3.8)^D^	(12.1±5.2)^DE^	(13.8±1.8)^a^
	Chicken	(2.7±1.6)^F^	(9.1±3.3)^E^	(13.6±3.0)^DE^	(8.5±5.4)^b^
	Average	(8.2±6.1)^b^	(12.4±4.8)^a^	(12.8±3.9)^a^	
C20:1 ω-9	Beef	(3.0±0.8)^D^	(4.0±1.4)^D^	(0.1±0.1)^E^	(2.3±2.0)^a^
	Chicken	(0.0±0.0)^E^	(0.4±0.2)^e^	(0.6±0.1)^E^	(0.3±0.3)^b^
	Average	(1.5±1.7)^a^	(2.2±2.2)^a^	(0.3±0.3)^b^	
C21:0	Beef	(0.0±0.0)^E^	(0.0±0.0)^E^	(1.6±0.4)^D^	(0.5±0.9)^a^
	Chicken	(0.0±0.0)^E^	(0.4±0.1)^E^	(0.0±0.0)^E^	(0.1±0.2)^b^
	Average	(0.0±0.0)^b^	(0.2±0.2)^b^	(0.8±0.9)^a^	
C22:0	Beef	(0.0±0.0)^F^	(0.0±0.0)^F^	(0.0±0.0)^F^	(0.0±0.0)^b^
	Chicken	(4.4±0.9)^D^	(1.1±0.2)^E^	(1.0±0.8)^E^	(2.2±1.9)^a^
	Average	(2.2±2.5)^a^	(0.6±0.6)^b^	(0.5±0.8)^b^	
C20:3 ω-3	Beef	(1.3±1.1)	(1.3±1.3)	(0.1±0.2)	(0.9±0.7)^a^
	Chicken	(0.0±0.0)	(0.0±0.0)	(0.0±0.0)	(0.0±0.0)^b^
	Average	(0.6±1.0)	(0.6±1.1)	(0.0±0.1)	
C22:1 ω-9	Beef	(0.0±0.0)^E^	(0.0±0.0)^E^	(0.8±0.7)^D^	(0.3±0.5)
	Chicken	(0.0±0.0)^E^	(0.0±0.0)^E^	(0.0±0.0)^E^	(0.0±0.0)
	Average	(0.0±0.0)^b^	(0.0±0.0)^b^	(0.4±0.6)^a^	
C23:0	Beef	(5.4±0.8)	(7.3±2.4)	(3.3±2.2)	(5.3±2.0)^a^
	Chicken	(0.0±0.0)	(0.1±0.2)	(0.5±0.2)	(0.2±0.2)^b^
	Average	(2.7±3.0)	(3.7±4.2)	(1.9±2.1)	
C22:2 ω-6	Beef	(0.0±0.0)^E^	(0.0±0.0)^E^	(0.2±0.2)^D^	(0.1±0.1)
	Chicken	(0.0±0.0)^E^	(0.0±0.0)^E^	(0.00±0.00)^E^	(0.0±0.0)
	Average	(0.0±0.0)^b^	(0.0±0.0)^b^	(0.1±0.2)^a^	
C24:0	Beef	(2.0±0.3)^E^	(2.9±1.0)^D^	(1.1±0.7)^F^	(2.0±0.9)^a^
	Chicken	(0.0±0.1)^G^	(0.0±0.1)^G^	(0.05±0.08)^G^	(0.0±0.0)^b^
	Average	(1.0±1.1)^ab^	(1.5±1.7)^a^	(0.6±0.7)^b^	
C24:1 ω-9	Beef	(0.0±0.0)^E^	(0.0±0.0)^E^	(0.4±0.4)^D^	(0.2±0.3)
	Chicken	(0.1±0.2)^E^	(0.0±0.0)^E^	(0.0±0.0)^E^	(0.0±0.1)
	Average	(0.0±0.1)	(0.0±0.0)	(0.2±0.4)	
C22:6 ω-3	Beef	(2.0±0.7)	(3.4±1.0)	(2.6±1.8)	(2.7±0.7)^a^
	Chicken	(0.2±0.1)	(0.0±0.0)	(0.3±0.0)	(0.2±0.2)^b^
	Average	(1.0±1.5)	(1.7±2.0)	(1.5±1.7)	
SFA	Beef	(17.1±5.0)	(15.8±3.6)	(12.8±3.2)	(15.2±2.2)^b^
	Chicken	(44.5±4.9)	(29.5±17.5)	(19.9±16.3)	(31.3±12.4)^a^
	Average	(30.8±15.6)	(22.7±13.6)	(16.4±11.2)	
MUFA	Beef	(40.6±2.3)	(38.7±3.7)	(41.9±9.2)	(40.4±1.6)^a^
	Chicken	(8.9±0.6)	(17.7±14.9)	(25.8±15.0)	(17.5±8.4)^b^
	Average	(24.7±17.4)	(28.2±15.0)	(33.9±14.2)	
PUFA	Beef	(42.3±2.8)	(45.5±0.9)	(45.2±6.2)	(44.3±1.8)^b^
	Chicken	(46.6±5.4)	(52.6±2.5)	(54.4±3.5)	(51.2±4.1)^a^
	Average	(44.5±4.5)^b^	(49.1±4.3)^a^	(49.8±6.8)^a^	
ω-3	Beef	(3.2±2.8)	(4.7±1.6)	(2.7±1.7)	(3.6±1.0)^a^
	Chicken	(0.2±0.1)	(0.00±0.00)	(0.3±0.0)	(0.2±0.2)^b^
	Average	(1.7±2.4)	(2.4±2.8)	(1.5±1.7)	
ω-6	Beef	(13.7±0.2)	(15.6±3.8)	(12.3±5.3)	(13.9±1.6)^a^
	Chicken	(2.7±1.6)	(9.1±3.3)	(13.6±3.0)	(8.5±5.4)^b^
	Average	(8.2±6.1)	(12.4±4.8)	(13.0±3.9)	
ω-9	Beef	(16.7±3.8)	(14.2±2.7)	(15.6±6.3)	(15.5±1.2)^a^
	Chicken	(8.5±0.4)	(8.4±1.0)	(7.9±0.4)	(8.3±0.3)^b^
	Average	(12.6±5.1)	(11.3±3.7)	(11.7±5.8)	

Mean values in the same column/row with different lowercase letters in superscript differ significantly (p<0.01) within individual treatments. Mean values in the same column/row with different uppercase letters in superscript indicate significant differences due to interaction effects between types of meat and mass fractions of tuna as a meat substitute (p<0.01). Results are expressed as mean value±standard deviation, *N*=5

As many as 27 types of fatty acids were detected in the meatballs made from beef that was partially replaced with tuna. The partial substitution of meatballs with tuna increased the PUFA content, with the strongest effect observed in chicken meatballs containing 40 % tuna. This increase is due to the high PUFA content in both chicken and tuna meat (Table 1). The high PUFA content in chicken meatballs partially replaced with tuna is primarily due to the increased content of C18:2 ω-6 (methyl linolelaidate). Untea *et al.* (*35*) reported that the C18:2 ω-6 content in chicken fed with walnut meal and cranberry leaves ranged from 26.81 to 32.33 %.

Increasing the mass fraction of tuna added to other types of meat generally decreased the SFA mass fraction, while increasing the MUFA, PUFA and ω-6 mass fractions in the meatballs, although these changes were not statistically significant (Table 6). The decrease in SFA content was due to lower mass fractions of C16:0 (methyl palmitate) and C22:0 (methyl decanoate). Conversely, the increase in MUFA was associated with higher mass fractions of C15:1 (methyl *cis*-10-pentadecanoate), C16:1 (methyl palmitoleate), C17:1 (methyl *cis*-10-heptadecenoate) and C22:1 ω-9 (methyl erucate). The increase in PUFA and ω-6 mass fractions was associated with an increase of C18:3 ω-6 (γ-linolenic acid methyl ester) and C22:2 ω-6 (methyl *cis*-13,16-docosadienoic acid) mass fractions. Meatballs made from beef with tuna substitute had increased mass fractions of MUFA, ω-3, ω-6 and ω-9 (p<0.01), which was due to an increase in C15:1, C16:1, C17:1, C18:1 ω-9, C22:1 ω-9, C22:2 ω-6 and C24:1 ω-9 (methyl nervonate) content. Conversely, chicken meatballs with tuna substitute had higher SFA and PUFA mass fractions (p<0.01), with the increased SFA mass fraction due to increased C16:0, C17:0 (methyl heptadecanoate) and C22:0 (methyl docosanoate) content, and higher PUFA mass fractions due to increased C18:2 ω-6 (methyl linolelaidate) content.

### Sensory properties of meatballs partially replaced with tuna meat

Table 7 shows the sensory properties of the beef and chicken meatballs that were replaced with tuna meat. The analysis showed a significant (p<0.01) effect of tuna content on the colour of the meatballs, but no significant differences in aroma, elasticity, texture or taste. Meatballs with 40 % tuna meat substitute (P2) had a significantly higher colour score (4.1, grey) than those with 20 % tuna meat substitute (P1) (3.0, slightly grey) and those without tuna substitute (P0) (2.6, white, tending to slightly grey). The colour scores for meatballs with 40 % tuna substitute ranged from 4.0 to 4.1 (grey) for beef and chicken, while the lowest score of 1.7 (very white, tending to white) was found for chicken meatballs without tuna substitute.

**Table 7:** Sensory properties of with partial replacement of meat with tuna

		*w*(tuna)/%			
Parameter	Meat	0	20	40	Average
Colour	Beef	(3.5±0.7)^DE^	(3.1±1.0)^E^	(4.0±0.4)^D^	(3.5±0.5)^a^
	Chicken	(1.7±1.0)^F^	(3.0±0.8)^E^	(4.1±0.7)^D^	(3.0±1.2)^b^
	Average	(2.6±1.3)^b^	(3.03±0.05)^b^	(4.07±0.09)^a^	
Aroma	Beef	(3.8±0.9)	(2.9±0.9)	(2.9±1.1)	(3.2±0.5)
	Chicken	(3.3±1.3)	(3.3±0.7)	(3.3±1.0)	(3.31±0.04)
	Average	(3.6±0.3)	(3.1±0.3)	(3.1±0.3)	
Elasticity	Beef	(2.9±0.8)	(3.3±1.2)	(3.4±1.1)	(3.2±0.3)
	Chicken	(3.1±0.8)	(3.5±0.8)	(3.4±1.0)	(3.4±0.2)
	Average	(3.0±0.2)	(3.4±0.1)	(3.4±0.0)	
Texture	Beef	(2.6±0.7)	(3.1±1.0)	(3.1±0.7)	(2.9±0.3)^b^
	Chicken	(3.5±0.8)	(3.4±0.7)	(3.7±1.2)	(3.5±0.2)^a^
	Average	(3.0±0.6)	(3.2±0.2)	(3.4±0.5)	
Taste	Beef	(3.4±0.6)	(3.0±1.1)	(3.3±0.9)	(3.2±0.2)^b^
	Chicken	(3.8±1.1)	(3.6±0.7)	(4.1±0.8)	(3.8±0.2)^a^
	Average	(3.6±0.3)	(3.3±0.4)	(3.7±0.5)	

Mean values in the same column/row with different lowercase letters in superscript differ significantly (p<0.01) within individual treatments. Mean values in the same column/row with different uppercase letters in superscript indicate significant differences due to interaction effects between types of meat and mass fractions of tuna as a meat substitute (p<0.01). Results are expressed as mean value±standard deviation, *N*=5

The type of meat significantly influenced the texture and taste of the meatballs. Chicken meatballs with tuna substitutes had a higher texture score of 3.5 (rather smooth, tending to be smooth), which was significantly higher (p<0.01) than the texture score of 2.9 (rough, tending to be rather smooth) for beef meatballs with tuna substitutes. The taste of chicken meatballs was also rated 3.8 (rather tasty, tending to be tasty) and was thus significantly higher (p<0.01) than the score of 3.2 (rather tasty) for beef meatballs with tuna substitute.

The colour of the meatballs is primarily affected by the type of meat used and its myoglobin content, as well as by fillers such as flour. A higher mass fraction of tuna replacing meat improved the colour of the meatballs, resulting in scores between 4.0 and 4.1 (grey). This result is consistent with the findings of Hetharia *et al.* (*36*), who found that replacing pork in beef meatballs resulted in a grey colour due to the lower myoglobin content of pork than of beef.

Chicken meatballs replaced with tuna had a better texture and taste than those made with beef. The chicken meatballs achieved a texture score of 3.5 (slightly smooth, tending to be smooth) and a taste score of 3.8 (rather tasty, tending to be tasty). The improved texture is due to the finer muscle fibre in chicken, as noted by Weng *et al.* (*37*), who reported that rapidly growing broiler chickens have thinner muscle fibres.

Taste plays a crucial role in consumer acceptance of meatball products and is influenced by characteristics such as aroma and colour (*35*). The acceptability of meatballs is often determined by key flavours such as savory, salty and meaty. The preference for chicken meatballs with tuna substitutes could be related to their more appealing aroma, although this difference was not statistically significant.

## CONCLUSIONS

Increasing the mass fraction of tuna as a substitute for beef and chicken has a positive effect on the chemical and sensory quality of the meatballs. This replacement leads to a higher moisture and protein content, a higher mass fraction of polyunsaturated fatty acids (PUFAs) and better colour, while the fat and cholesterol content of the meatballs is reduced. Notably, replacing chicken meat with 40 % tuna leads to an increase in fat content. Although not statistically significant, replacing beef and chicken with tuna leads to a decrease in saturated fatty acids (SFAs) and an increase in monounsaturated fatty acids (MUFAs).

The study shows that replacing beef with 40 % tuna has more pronounced benefits, such as higher moisture, protein and PUFA content and better colour, while reducing fat and cholesterol. Conversely, replacing chicken with 40 % tuna results in a higher fat content in the meatballs.

These results have significant implications for consumers, meat producers and scientific research. For consumers, this substitution can reduce health risks associated with meatballs, as the products are richer in protein and lower in fat and cholesterol. For meat producers, the knowledge gained can be used to improve the quality of other processed meat products, such as sausages and nuggets. For scientific research, this study provides valuable contributions in the field of processed meat and shows possibilities for further development of high-quality healthier meat products.
